# Potential of Murine IgG1 and Human IgG4 to Inhibit the Classical Complement and Fcγ Receptor Activation Pathways

**DOI:** 10.3389/fimmu.2018.00958

**Published:** 2018-05-09

**Authors:** Gina-Maria Lilienthal, Johann Rahmöller, Janina Petry, Yannic C. Bartsch, Alexei Leliavski, Marc Ehlers

**Affiliations:** ^1^Laboratories of Immunology and Antibody Glycan Analysis, Institute for Nutrition Medicine, University of Lübeck and University Medical Center of Schleswig-Holstein, Lübeck, Germany; ^2^Department of Anesthesiology and Intensive Care, University of Lübeck and University Medical Center of Schleswig-Holstein, Lübeck, Germany; ^3^Airway Research Center North (ARCN), University of Lübeck, German Center for Lung Research (DZL), Lübeck, Germany

**Keywords:** complement, C1q, IgG4, IgG, IgG hexamer, IgG glycosylation, immunosuppression, murine IgG1

## Abstract

IgG antibodies (Abs) mediate their effector functions through the interaction with Fcγ receptors (FcγRs) and the complement factors. The main IgG-mediated complement activation pathway is induced through the binding of complement C1q to IgG Abs. This interaction is dependent on antigen-dependent hexamer formation of human IgG1 and IgG3 to increase the affinity for the six-headed C1q molecule. By contrast, human IgG4 fails to bind to C1q. Instead, it has been suggested that human IgG4 can block IgG1 and IgG3 hexamerization required for their binding to C1q and activating the complement. Here, we show that murine IgG1, which functionally resembles human IgG4 by not interacting with C1q, inhibits the binding of IgG2a, IgG2b, and IgG3 to C1q *in vitro*, and suppresses IgG2a-mediated complement activation in a hemolytic assay in an antigen-dependent and IgG subclass-specific manner. From this perspective, we discuss the potential of murine IgG1 and human IgG4 to block the complement activation as well as suppressive effects of sialylated IgG subclass Abs on FcγR-mediated immune cell activation. Accumulating evidence suggests that both mechanisms seem to be responsible for preventing uncontrolled IgG (auto)Ab-induced inflammation in mice and humans. Distinct IgG subclass distributions and functionally opposite IgG Fc glycosylation patterns might explain different outcomes of IgG-mediated immune responses and provide new therapeutic options through the induction, enrichment, or application of antigen-specific sialylated human IgG4 to prevent complement and FcγR activation as well.

## Introduction

IgG antibodies (Abs) mediate their effector functions through the interaction with Fcγ receptors (FcγRs) and the complement system. The different IgG subclasses thereby differ by their specificities and affinities to the classical activating and inhibitory FcγRs (1–5). Furthermore, the effector function of IgG Abs depends on the type of IgG Fc glycosylation pattern. Non-(a)galactosylated IgG Abs are associated with pro-inflammatory functions, such as IgG autoAbs in rheumatoid arthritis patients, whereas galactosylated and sialylated IgG Abs have reduced inflammatory and even immunosuppressive potential (6–19). IgG Fc sialylation reduces the affinity to the classical FcγRs and, instead, promotes interaction with members of the sugar-binding C-type lectin receptor family (6–8, 13, 14, 19).

In the context of the complement activation, IgG Abs of different subclasses show highly diverse affinities to the complement C1q molecule, which initiates the classical complement pathway (3, 20–23).

Antigen-dependent hexamer formation of six monomeric human IgG1 or IgG3 Abs through non-covalent Fc:Fc interactions, such as polymeric IgM, favors the interaction with one six-headed C1q molecule leading to complement activation (22–27). Upon hexamerization, only one Fab arm of each IgG Ab interacts with the antigen. The other Fab arm is positioned in the plane of the Fc hexamer (22, 23).

By contrast, human IgG4 fails to activate the complement through C1q binding (22, 25). It has been suggested that human IgG4 might still be able to form hexamers, but subclass-specific sequence differences affect orientation of the loops in the CH2 domain, which in turn alters the position of the C1q-binding site and therewith prevents the interaction with C1q (3, 28).

On the other hand, human IgG4 as well as IgA might prevent the hexamer formation of human IgG1 and IgG3 and ultimately C1q binding by steric interference (25, 28–32).

To explore the extent of this suggested inhibition, we studied whether murine IgG1, which functionally resembles human IgG4 by not interacting with C1q, can prevent the interaction of the murine C1q-binding IgG2a, IgG2b, and IgG3 subclasses with C1q and therefore block the complement activation.

We further discuss the potential of murine IgG1 and human IgG4 to inhibit the complement activation as well as how different IgG Fc glycosylation patterns and IgG subclasses affect IgG Fc receptor interactions during IgG-mediated immune responses, which may be exploited for development of novel therapeutic strategies.

## Materials and Methods

### Reagents

For the experiments described below, 2,4,6-trinitrophenyl (TNP)-coupled Ficoll (TNP-Ficoll) was purchased from Biosearch Technologies (Petaluma, CA, USA). 2,4,6-trinitrobenzenesulfonic acid or picrylsulfonic acid (TNBS)-solution was purchased from Sigma-Aldrich (St. Louis, MO, USA).

### Production of Monoclonal IgG Abs

The TNP-specific monoclonal hybridoma IgG1 (clone H5) (10, 11, 19, 33, 34) and IgG1, IgG2a, and IgG2b class-switch variant (sv; with identical VDJ sequences) (19) Abs as well as the ovalbumin (OVA)-specific IgG1 (clone 4C9) (10) Abs were produced, purified and verified as described (19).

### C1q ELISA

96-well ELISA plates were coated with 10 µg of TNP-Ficoll per milliliter overnight and subsequently incubated with the indicated combinations of monoclonal anti-TNP IgG subclass Abs in PBS containing 0.1% TWEEN 20 (Sigma-Aldrich). Unbound Abs were removed with PBS containing 0.05% TWEEN 20, and wells were incubated with human serum from healthy voluntary donors diluted 1:20 in HBSS with Ca^2+^ and Mg^2+^ (Gibco^®^ by LifeTechnologies, Paisley, UK) containing 0.1% TWEEN 20 for 1 h at room temperature. Unbound serum C1q was removed with PBS containing 0.05% TWEEN 20, and bound C1q was detected with HRP-labeled polyclonal sheep anti-human C1q IgG Abs (AbD Serotec, Bio-Rad, UK) and TMB substrate (BD Bioscience San Diego, CA, USA) at OD 450 nm. As a negative control, unspecific serum C1q binding was detected in TNP-Ficoll-coated wells without anti-TNP IgG Abs (OD at 450 nm was below 0.09).

### Preparation of TNP-Labeled Red Blood Cells (RBCs)

2,4,6-Trinitrophenyl labeling of human RBCs from healthy voluntary donors was performed in cacodylate buffer (pH 6.9) containing 0.4% TNBS-solution (both from Sigma-Aldrich) for 1 h at room temperature. Subsequently, the cells were centrifuged, resuspended in PBS containing 1 mg/ml of glycylglycine (Sigma-Aldrich) and washed with PBS. TNP-coupled RBCs were stabilized in citrate-phosphate-dextrose solution containing adenine (Sigma-Aldrich) and stored up to 4 weeks at 4°C. TNP-coupling to RBCs was verified by flow cytometry with murine anti-TNP IgG2a (sv) and FITC-labeled anti-murine IgG2a (Bethyl Laboratories, Montgomery, TE, USA) Abs (Figure S1E in Supplementary Material).

### Hemolysis Assay

2,4,6-Trinitrophenyl-coupled RBC suspensions (10^9^ cells/ml) were prepared as described (35). 50 µl of cells was sensitized with the indicated concentrations and combinations of one or two monoclonal anti-TNP IgG subclass Abs for 30 min at 37°C. To avoid unwanted agglutination, the highest non-agglutinating concentration was determined (22.5 µg/ml for IgG2a) (data not shown). Unbound Abs were removed by washing the samples first with GVB° and then GVB^++^ (with Ca^2+^ and Mg^2+^) buffer (ComplementTechnology, Tyler, TX, USA). Anti-TNP IgG subclass Ab-sensitized TNP-labeled RBCs were incubated with human serum from healthy volunteers diluted 1:3 in GVB^++^ buffer to induce complement-mediated RBC lysis. Complement activation and RBC lysis were stopped after 30 min at 37°C by adding 1 ml of ice cold 0.9% NaCl. Samples were centrifuged at 2,000 *g* for 5 min at 4°C and free hemoglobin in the supernatant was measured at 414 nm. The degree of RBC lysis (in %) was set in relation to a positive control lysis with H_2_O (100%). As a negative control, non-IgG sensitized TNP-coupled RBCs were incubated with the same serum-GVB^++^ preparation resulting in less than 4% lysis compared with the H_2_O-induced lysis. The induction of the classical complement pathway in this lysis assay was verified by blocking Ca^2+^ with EGTA (data not shown).

### Statistical Analysis

Statistical analyses were performed using GraphPad Prism software. Student’s *t*-test was used for comparing two groups or one-way ANOVA for comparing more than two groups: *P*-values below 0.05 were considered statistically significant (*P < 0.05, **P < 0.01, and ***P < 0.001). EC50 values were calculated using a non-linear regression with the variable slope model.

## Results

### Murine IgG1 Prevents Complex Formation of IgG2a, IgG2b, and IgG3 With C1q

**Figure 1:**
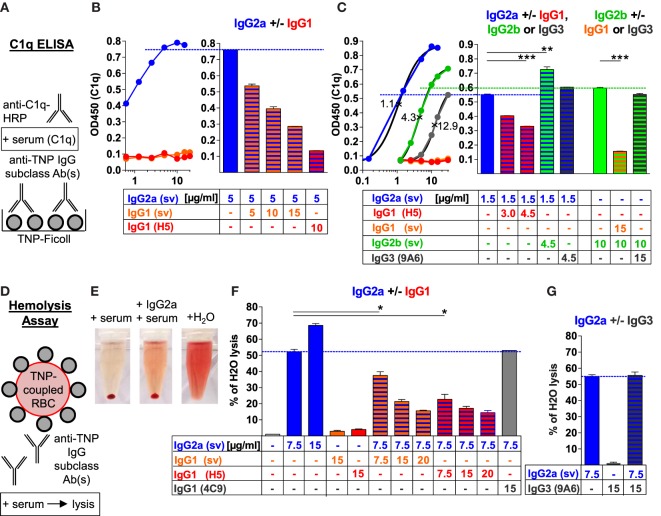
Murine IgG1 prevents C1q binding and complement activation by IgG2a, IgG2b, or IgG3 antibodies (Abs) in an antigen-specific manner. **(A)** Schematic description of the C1q ELISA applied in panels **(B,C)** and Figure S1 in Supplementary Material. 2,4,6-Trinitrophenyl (TNP)-Ficoll-coupled 96-well plates were incubated with different concentrations of one or two anti-TNP monoclonal IgG subclass Abs [anti-TNP IgG1 (clone H5; red), IgG3 (clone 9A6; gray) as well as IgG1 (orange), IgG2a (blue), and IgG2b (green) class-switch variants (sv; with identical VDJ sequences)] and subsequently with C1q-containing serum; C1q was detected with an anti-C1q-HRP-coupled secondary Ab system. **(B,C)** Mean of the resulting C1q ELISA values measured at 450 nm (OD450) with the indicated (μg/ml) single or paired anti-TNP IgG subclass Abs (*n* = 2). IgG subclass-specific half-maximal effective concentrations (EC50; ×) were calculated by the interpolated subclass-specific C1q-binding curves in panel **(C)** (*R*^2^ > 0.99). **(D)** Schematic description of the applied red blood cell (RBC)-lysis assay. TNP-coupled RBCs (Figure S1E in Supplementary Material) were incubated with one or two anti-TNP monoclonal IgG subclass Abs or an anti-ovalbumin IgG1 (clone 4C9) Ab and subsequently treated with serum containing C1q and further complement components. **(E)** Exemplary hemolysis approach with centrifuged TNP-coupled RBCs after reaction with serum, serum plus anti-TNP IgG2a or H_2_O as a positive control (100% lysis). **(F,G)** Mean of the resulting RBC lysis [measured hemoglobin (OD 414 nm) in the supernatant of centrifuged RBCs], which was calculated as the percentage of H_2_O-induced positive control RBC lysis (100%; maximum of the *y*-axes) with the indicated (μg/ml) single or paired IgG subclass Abs (*n* = 2). The results from one of at least two independent experiments are presented.

To investigate antigen-dependent interference between non-C1q-binding murine IgG1 and C1q-binding IgG2a, IgG2b, and IgG3 subclasses for C1q binding, we used TNP-specific murine monoclonal IgG1 (clone H5), IgG3 (clone 9A6), and IgG1, IgG2a, and IgG2b class-switch variant (sv; with identical VDJ sequences) Abs (10, 19, 33, 34, 36, 37). Incubation of plate-bound TNP-Ficoll with different concentrations of single TNP-specific IgG subclasses and serum as a source of C1q showed preferential binding of C1q to IgG2a (sv), but also to IgG2b (sv) and IgG3 (9A6). The calculated half-maximal effective concentrations (EC50) of the interpolated IgG subclass-specific C1q-binding curves in this approach were 1.1 µg/ml for IgG2a (sv), 4.3 µg/ml for IgG2b (sv), and 12.5–12.9 µg/ml for IgG3 (9A6), although the EC50 value for IgG3 could not directly be compared with the values for the switch variants IgG2a and IgG2b due to differences in their VDJ sequences (Figures 1A–C; Figure S1 in Supplementary Material). The two monoclonal IgG1 clones (sv and H5) failed to interact with C1q in this approach (Figures 1A–C).

However, the combination of two different IgG subclasses showed that both IgG1 clones inhibited the binding of C1q to IgG2a, IgG2b, and IgG3 in a concentration-dependent manner (Figures 1A–C; Figure S1 in Supplementary Material). The IgG1 clone H5 showed a stronger inhibition than the IgG1 sv clone because of a higher affinity to TNP (measured by affinity ELISA; data not shown).

At an almost saturated C1q-binding dosage of IgG2a (5 µg/ml), C1q binding was inhibited about 2- or 10-fold by a 2-fold amount of the IgG1 sv or using IgG1 clone H5 of higher affinity, respectively (Figure 1B; Figure S1A in Supplementary Material). At an IgG2a concentration (1.5 µg/ml) near its EC50, C1q binding to IgG2a was inhibited about 1.4-fold by a 2-fold amount of the IgG1 clone H5 (Figure 1C; Figure S1B in Supplementary Material). At an almost saturated C1q-binding dosage of IgG2b (10 µg/ml), C1q binding was downregulated about 4- or 9-fold by only 1.5-fold amount of the IgG1 sv or the IgG1 clone H5, respectively (Figure S1C in Supplementary Material). At IgG3 concentrations (12 or 16 µg/ml) near its EC50 value, C1q binding was even inhibited threefold to fourfold by adding only half the dose of the IgG1 clone H5 (Figure S1D in Supplementary Material).

In summary, the weaker was the C1q-binding potential of an IgG subclass, the more efficient was the inhibition by the IgG1 Abs and the higher was the concentration of IgG2a, IgG2b, or IgG3, the stronger was the dose-dependent C1q-binding inhibition by IgG1.

By contrast, a combination of IgG2a with IgG2b further enhanced C1q binding compared with IgG2a alone and a combination of IgG2a with IgG3 (at a concentration at which IgG3 showed almost no C1q binding) or IgG2b with IgG3 did not reduce C1q binding compared with IgG2a or IgG2b alone (Figure 1C; Figure S1A–D in Supplementary Material). Thus, the murine IgG1 subclass exclusively inhibited binding of C1q to murine IgG2a, IgG2b, and IgG3.

### Murine IgG1 Prevents C1q-Mediated Complement Activation by IgG2a

Next, we investigated *in vitro* whether murine IgG1 can also inhibit complement-mediated lysis of RBCs induced by the other murine IgG subclasses. TNP was conjugated to purified RBCs (Figure S1E in Supplementary Material), and these cells were incubated with one murine IgG subclass or a combination of two different IgG subclasses. Serum was used as a source of C1q and further complement components to induce RBC lysis and hemoglobin release (Figures 1D–G). Complement-mediated RBC lysis was calculated as the percentage of complete lysis of the same amount of cells with H_2_O (set 100%; the positive control) (Figures 1E–G).

IgG2a alone induced a dose-dependent C1q-mediated RBC lysis when applied in the concentration range between 2.5 and 22.5 µg/ml (the highest used non-agglutinating concentration) and reached up to 70% of positive control H_2_O lysis (Figures 1E–G and data not shown). IgG2b alone poorly induced RBC lysis in the concentration range between 7.5 and 22.5 µg/ml and reached no more than 8% of positive control H_2_O lysis (data not shown). Murine IgG1 and also IgG3 alone barely induced any RBC lysis in this setting (1–4% of positive control H_2_O lysis), which was in the range of the negative control, when no IgG applied (Figures 1F,G and data not shown). Because IgG2b and IgG3 failed to induce significant RBC lysis, we further tested how IgG1 affects IgG2a-mediated RBC lysis only.

Both IgG1 clones inhibited IgG2a-induced RBC lysis. The IgG1:IgG2a ratio of 2:1 led to a 2.4- or 3-fold inhibition of the IgG2a-mediated lysis with the IgG1 sv or the IgG1 clone H5, respectively. By contrast, IgG2b enhanced IgG2a-induced RBC lysis, whereas IgG3 had no effect (Figures 1F,G and data not shown). Furthermore, the IgG1-mediated complement inhibition was antigen-specific, because antigen-unspecific, OVA-specific murine IgG1 failed to inhibit the IgG2a-mediated RBC lysis (Figure 1F).

Thus, the murine IgG1 subclass exclusively inhibited IgG2a- and C1q-dependent complement activation in the hemolysis assay, in an antigen-specific manner.

## Discussion

### Murine IgG1 and Human IgG4 May Inhibit Hexamer Formation of the Other IgG Subclasses and Consequently Their C1q Binding by Steric Interference

**Figure 2:**
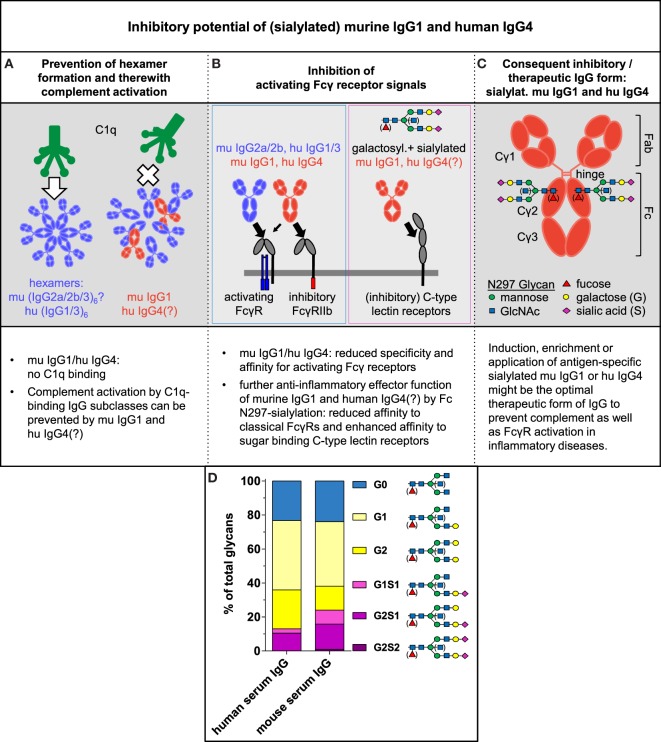
Inhibitory potential of (sialylated) murine IgG1 and human IgG4. **(A–C)** Summary of our perspective about the inhibitory and therapeutic potential of (sialylated) murine IgG1 and human IgG4 (see text for details). IgG antibodies have one conserved *N*-glycosylation site at Asn 297 in each of their constant heavy chain regions. The biantennary core glycan structure that consists of four *N*-acetylglucosamine (GlcNAc) and three mannose residues can be further decorated with fucose, a bisecting GlcNAc and terminal galactose or galactose and sialic acid. **(D)** Average distribution of total human and mouse serum IgG Fc glycosylation patterns coupled to Asn 297 of total human serum IgG (IVIg; pooled intravenous immunoglobulin from healthy donors) and total murine serum IgG from 8- to 10-week-old untreated female C57BL/6 wild-type mice were analyzed by high-pressure liquid chromatography [data from Epp et al. (19)].

Human IgG1 and IgG3 Abs form hexamers *via* non-covalent Fc:Fc interactions in an antigen-specific manner to bind C1q. Although it has not been determined whether the murine IgG2a, IgG2b, and IgG3 Ab subclasses can also form hexamers to bind C1q and activate the classical complement pathway, we showed that murine IgG1 can prevent the interaction of C1q-binding murine IgG subclasses with C1q, as suggested for human IgG4 (25). Dampening of C1q binding by murine IgG1 or human IgG4 might result from competition for antigen binding and/or from steric interference between antigen-bound murine IgG1 or human IgG4 and the other IgG subclasses to inhibit hexamer formation of the latter Abs (25) (Figure 2A). The potential binding of murine IgG1 or human IgG4 to an antigen in close vicinity to the C1q-binding IgG subclasses might interfere with Fc:Fc contact formation, potentially leading to reduced hexamerization, C1q binding, and complement activation (23, 25). Efficient steric interference may be dependent on antigen density and epitope specificity, as well as on affinity of murine IgG1 and human IgG4.

Human IgG4 exploits additional properties to modify immune responses, such as dynamic Fab-arm exchange, which results in formation of bispecific IgG4 Abs (38, 39), and the interaction with the Fc part of other IgG subclass Abs (40–45). Both mechanisms might also contribute to the human IgG4-mediated suppression of the hexamer formation by other IgG subclasses; but it has not been yet demonstrated for murine IgG1.

The possibility that antigen-dependent steric interference by murine IgG1 or human IgG4 prevents the hexamer formation by other IgG subclasses might suggest a scenario, in which a single murine IgG1 or human IgG4 molecule is sufficient to reduce the hexamer formation of the other IgG subclass molecules, conferring anti-inflammatory properties to murine IgG1 or human IgG4 *via* blocking the complement activation.

### Anti-Inflammatory Functions of Murine IgG1 and Human IgG4 in the Context of FcγR Interactions

Fcγ receptor-mediated effector functions vary between IgG subclasses and depend on FcγR distribution on the surface of myeloid and lymphoid immune cells. Murine IgG1, as well as human IgG4, show limited specificities and affinities to the classical activating FcγRs and preferably interact with the classical IgG inhibitory receptor FcγRIIb, as compared with murine IgG2a and IgG2b as well as human IgG1 and IgG3 (Figure 2B; Figure S2 in Supplementary Material) (1–5, 46, 47). Thus, both the IgG subclass distribution and the expression levels of classical activating and inhibitory FcγRs determine the balance between activating and inhibitory signals in the target cells.

Mechanistically, crosslinking of IgG Abs with classical activating FcγRs induces phosphorylation of their own or their associated Fc receptor gamma (FcRγ)-chain intracellular immunoreceptor tyrosine-based activation motif (ITAM) (Figure S2 in Supplementary Material) and activation of the target cell (Figure S2 in Supplementary Material) (2). However, additional IgG-mediated crosslinking of the inhibitory FcγRIIb can lead to the phosphorylation of its intracellular immunoreceptor tyrosine-based inhibition motif (ITIM) and thereby inhibition of cell activation (Figure S2 in Supplementary Material) (2). In general, the phosphorylation of an ITIM motif always depends on upstream ITAM phosphorylation, for example, here, of activating FcγR (Figure S2 in Supplementary Material) (2).

Moreover, FcγR-mediated pro- or anti-inflammatory effector functions of murine and human IgG Abs are effected by the IgG Fc *N*-glycosylation pattern at Asn 297 in the CH2 region. Enriched amounts of non-(a)galactosylated IgG (auto)Abs are associated with pro-inflammatory effector functions of the IgG Abs and (auto)immune disease severity, such as IgG autoAbs in patients with rheumatoid arthritis, whereas galactosylated and sialylated IgG Abs possess reduced inflammatory and even immunosuppressive potential (Figure 2D) (6–12, 14, 17–19, 34, 48). IgG Fc galactosylation and sialylation seems to alter the conformation of the Fc portion and ultimately shifts its affinity from the classical FcγRs toward the so-called type II FcRs that mediate inhibitory signaling. Several sugar-binding C-type lectin receptors belong to the type II FcRs, including murine specific ICAM-3-grabbing non-integrin-related 1 (SIGN-R1) and its human homolog dendritic cell-specific ICAM-3-grabbing non-integrin (DC-SIGN); CD209, murine dendritic cell inhibitory receptor (DCIR), and CD23 (Figure 2B; Figure S2 in Supplementary Material) (4, 7, 8, 12–15, 18, 19, 49–53).

SIGN-R1 and DC-SIGN have originally been identified as receptors that recognize carbohydrates of some bacteria, e.g., *S. pneumonia* (4, 7, 14, 51). SIGN-R1 and DC-SIGN contain neither an ITAM nor an ITIM motif and the induction of inhibitory signals by sialylated IgG Abs *via* these receptors is yet unclear (Figure S2 in Supplementary Material).

Dendritic cell inhibitory receptor interacts with glycans of both pathogenic and endogenous origins and has, in contrast to SIGN-R1/DC-SIGN, an intracellular ITIM motif (4, 13, 49, 50, 54). However, it is still unknown whether sialylated IgG immune complexes (ICs) crosslink DCIR with ITAM-containing classical FcγRs or other ITAM-containing receptors to deliver inhibitory signals (Figure S2 in Supplementary Material).

CD23 is known as the low-affinity IgE receptor present on some immune cells, including B lymphocytes. However, also sialylated IgG Abs can crosslink CD23, which leads to up-regulation of FcγRIIB expression (Figure S2 in Supplementary Material) (4, 14, 15, 55). The induced inhibitory signals by CD23 might be mediated by crosslinking of CD23 with the B cell receptor (BCR) and thereby modulating the BCR signal.

The structure of the IgG Fc glycan has different impact on the effector functions of the different IgG subclasses: it strongly influences functional properties of murine IgG1, less of IgG2b, but barely of IgG2a (10, 11, 19, 34, 48).

Recent studies have shown that sialylated antigen-specific murine IgG1 Abs can be used to induce antigen-specific immune tolerance, making such Abs a potential therapeutic tool to treat inflammatory immune diseases in an antigen-specific manner (10–12, 16).

In addition to the anti-inflammatory potential of sialylated murine IgG1, only galactosylation of murine IgG1 has also induced inhibitory signals in a mouse model of autoimmune hemolytic anemia and *via* crosslinking the C-type lectin receptor Dectin-1 (Clec7a) with the inhibitory receptor FcγRIIb (Figure S2 in Supplementary Material) (12, 34, 56). Dectin-1 recognizes bacterial carbohydrates and activates effector cells *via* an intracellular ITAM-like (hemITAM) motif (34, 51, 57). However, in proximity to FcγRIIb, the ITAM-like motif of Dectin-1 induces phosphorylation of the ITIM motif of FcγRIIb and promotes inhibitory signaling (34). Recently, it has further been suggested that the galactose-binding receptor galectin-3 is additionally involved in this complex and directly interacts with galactosylated murine IgG1 and induces complex formation between Dectin-1 and FcγRIIb (58). In humans, inhibitory effects of galactosylated IgG Abs have been described for the plasmacytoid dendritic cell-specific type II C-type lectin receptor blood dendritic cell antigen 2, which signals *via* the ITAM-containing FcRγ-chain as signaling molecule (59, 60).

It has still to be determined to which extent Fc glycosylation affects effector properties of human IgG molecules in a subclass-dependent manner, but the current suggestion is that the effector function of all human IgG subclasses depends on the Fc glycosylation pattern.

Interestingly, the hexamer formation of human IgG1 is dependent on the conserved IgG Fc glycosylation site (23) and human IgG1 Fc galactosylation favors C1q binding, as compared with agalactosylated as well as sialylated human IgG1 (61, 62).

Together, the observations mentioned before show that sialylated murine IgG1 and seemingly human IgG4 have an anti-inflammatory potential in the context of FcγR-mediated activation of the immune system.

### IgG Subclass Distribution and Fc Glycosylation Pattern Determine the Effector Function of IgG Abs, Making Sialylated Murine IgG1 and Human IgG4 Attractive Inhibitory (Therapeutic) Tools

In line with the above mentioned findings, it has recently been demonstrated that IgG1-deficient mice develop severe autoimmune conditions in different inflammatory autoimmune disease models (63, 64) as well as enhanced antigen-dependent IgG3 IC depositions in the kidneys (37). The autoimmune models used in these studies (63, 64), similar to other inflammatory autoimmune models, are apparently dependent on C1q and the complement activation as well as on classical FcγR-mediated immune cell activation (21, 61, 63–69). In future studies, it would be important to explore the inhibitory potential of murine IgG1 and its Fc glycosylation status in terms of the complement activation and FcγR-mediated regulation of effector cells using various models of autoimmunity and inflammation.

In summary, the mentioned results show that the analysis of the IgG subclass distribution and Fc glycosylation pattern during an immune response provides important information about the inflammatory state of the immune reaction. It is still a common practice that inflammatory and autoimmune diseases are diagnosed only by analysis of total (auto)antigen-specific IgG Abs. We believe that the additional analysis of IgG subclass distribution and their Fc glycosylation patterns will be essential for understanding the current inflammatory status of antigen-specific IgG-mediated immune response and associated pathologies. These data also suggest that murine IgG1 and human IgG4 Abs in particular might have the strongest inhibitory potential toward all other IgG forms, due to their possible inhibitory effect on both, complement and classical FcγR activation (Figure 2C).

Total IgG fraction in human serum mainly consists of human IgG1 and less of human IgG4 (IgG1 > IgG2 > IgG3 > IgG4). Changes in this distribution of IgG subclasses as well as in their Fc glycosylation patterns will presumably have a strong impact on IgG-mediated immune responses.

For many years, the induction of human IgG4 (for instance, induced by allergen-specific immunotherapies) has been associated with the inhibition of IgE-mediated allergic immune responses (19, 70). However, in the presence of high doses of allergen IgG Abs can also induce allergic reactions (19). In this context, an inhibitory role of human IgG4 has been discussed (19, 29). Furthermore, the MHC-specific human IgG1/3 to IgG4 ratio might be a critical parameter that determines the risk of complement-mediated transplant rejection in patients after organ transplantation and blood transfusion (71). More in-depth analysis of the IgG subclass distribution and Fc glycosylation might clarify inhibitory effects of human IgG4 in these studies.

Blockade of C1q with designer molecules has been proposed as a potent approach to treat inflammatory diseases. However, the shift of antigen-specific T and B cell responses to produce primarily sialylated human IgG4 or the application of enriched or *in vitro*-produced antigen-specific sialylated IgG4 Abs might be a promising alternative therapeutic approach to treat complement-mediated inflammatory diseases and to inhibit FcγR-mediated inflammatory responses at the same time. Recent studies have indeed shown for the first time that enrichment of type XVII collagen (Col17)-specific human IgG4 autoAbs from autoimmune patients with bullous pemphigoid skin disease and their administration to humanized Col17 mice inhibited the complement activation and disease development by interfering with the other IgG subclasses (72).

## Conclusion and Perspective

We showed that murine IgG1 inhibits C1q binding of IgG2a, IgG2b, and IgG3 and complement activation by murine IgG2a in an antigen-dependent manner, and suggested potential application of these findings for human IgG4. We further outline other inhibitory functions of murine IgG1 and human IgG4 and stressed the importance of future analysis of IgG subclass distribution and Fc glycosylation patterns in developing novel therapeutic approaches. We conclude that antigen-specific sialylated murine IgG1 and human IgG4 Abs might be a promising therapeutic tool to inhibit complement activation and abolish FcγR-mediated inflammatory responses in inflammatory (autoimmune) diseases.

## Ethics Statement

Human blood from healthy donors was collected with the approval of and in accordance with regulatory guidelines and ethical standards set by the University of Lübeck.

## Author Contributions

G-ML conducted the experiments. ME supervised the experiments and discussed and wrote the manuscript together with G-ML, JR, JP, YB, and AL.

## Conflict of Interest Statement

The authors declare that they have no commercial or financial relationships that could be construed as a potential conflict of interest.

## Supplementary Material

Figure S1 Inhibition of C1q binding to IgG2a, IgG2b, and IgG3 by IgG1. **(A–D)** C1q ELISA experiments as described and partially shown in Figures 1A–C with 2,4,6-trinitrophenyl (TNP)-Ficoll-coated 96-well plates, which were incubated with different concentrations of one or two anti-TNP monoclonal IgG subclass Abs [anti-TNP IgG1 (clone H5; red), IgG3 (clone 9A6; gray) as well as IgG1 (orange), IgG2a (blue), and IgG2b (green) class-switch variants (sv; with identical VDJ sequences)] and subsequently with serum containing C1q that was detected with an anti-C1q-HRP-coupled secondary Ab system. The left figure parts in panels **(A–D)** show the single IgG2a (blue), IgG2b (green), or IgG3 (gray) OD 450 nm (OD 450; left *y*-axes; each point: mean of *n* = 2) value binding curves and their interpolated binding curves with the percentage (right *y*-axes) of their maximal C1q binding [black; *R*^2^ of interpolation was >0.99; the top of the (interpolated) IgG subclass-specific curves was set to 100%, and the bottom of the curves was set to 0%]. The IgG subclass-specific half-maximal effective concentration (EC50) was calculated from the interpolated C1q-binding curves. To the indicated (dashed line) amounts of single IgG subclasses [**(A)** 5 µg/ml IgG2a; **(B)** 1.5 µg/ml IgG2a; **(C)** 10 µg/ml IgG2b; **(D)** 12 or 16 µg/ml IgG3], the indicated *x*-fold amounts of a second indicated anti-TNP IgG subclass were added. Their resulting OD 450 values and calculated percentages are presented in the right figure parts. **(E)** Verification of the TNP-coupling to red blood cell (RBC) as described in Figure 1D. TNP coupling to RBCs was verified by flow cytometry with murine anti-TNP IgG2a (sv) and FITC-labeled anti-murine IgG2a Abs. The results from one of at least two independent experiments are presented.

Figure S2 Expression of IgG Fc interacting receptors by human and murine immune effector cells and their immunoreceptor tyrosine-based activation motif (ITAM)/immunoreceptor tyrosine-based inhibition motif (ITIM) signaling motifs. List of type I and type II IgG Fc receptors as mentioned in the text. The expression pattern of the individual receptors on different immune cell types has been described before (1–5, 7, 8, 12–15, 18, 19, 34, 46, 47, 49–54, 57–60). Abbreviations: BC, B cells; TC, T cells; NK, natural killer cells; Mo, monocytes; MΦ, macrophages.

## Abbreviations

Ab, antibody; BCR, B cell receptor; BDCA-2, blood dendritic cell antigen 2; DC-SIGN, dendritic cell-specific ICAM-3-grabbing non-integrin; DCIR, dendritic cell inhibitory receptor; IC, immune complex; FcγR, Fcγ receptor; OVA, ovalbumin; RBC, red blood cell; sv, swich variant; SIGN-R1, specific ICAM-3-grabbing non-integrin-related 1; TNBS, 2,4,6-trinitrobenzenesulfonic acid or picrylsulfonic acid; TNP, 2,4,6-trinitrophenyl.
