# Supplementary figures and images for: Potential of Murine IgG1 and Human IgG4 to Inhibit the Classical Complement and Fcγ Receptor Activation Pathways
Source: Front Immunol. 2018 May 9;9:958. doi: 10.3389/fimmu.2018.00958 (PMC5954034; doi:10.3389/fimmu.2018.00958)

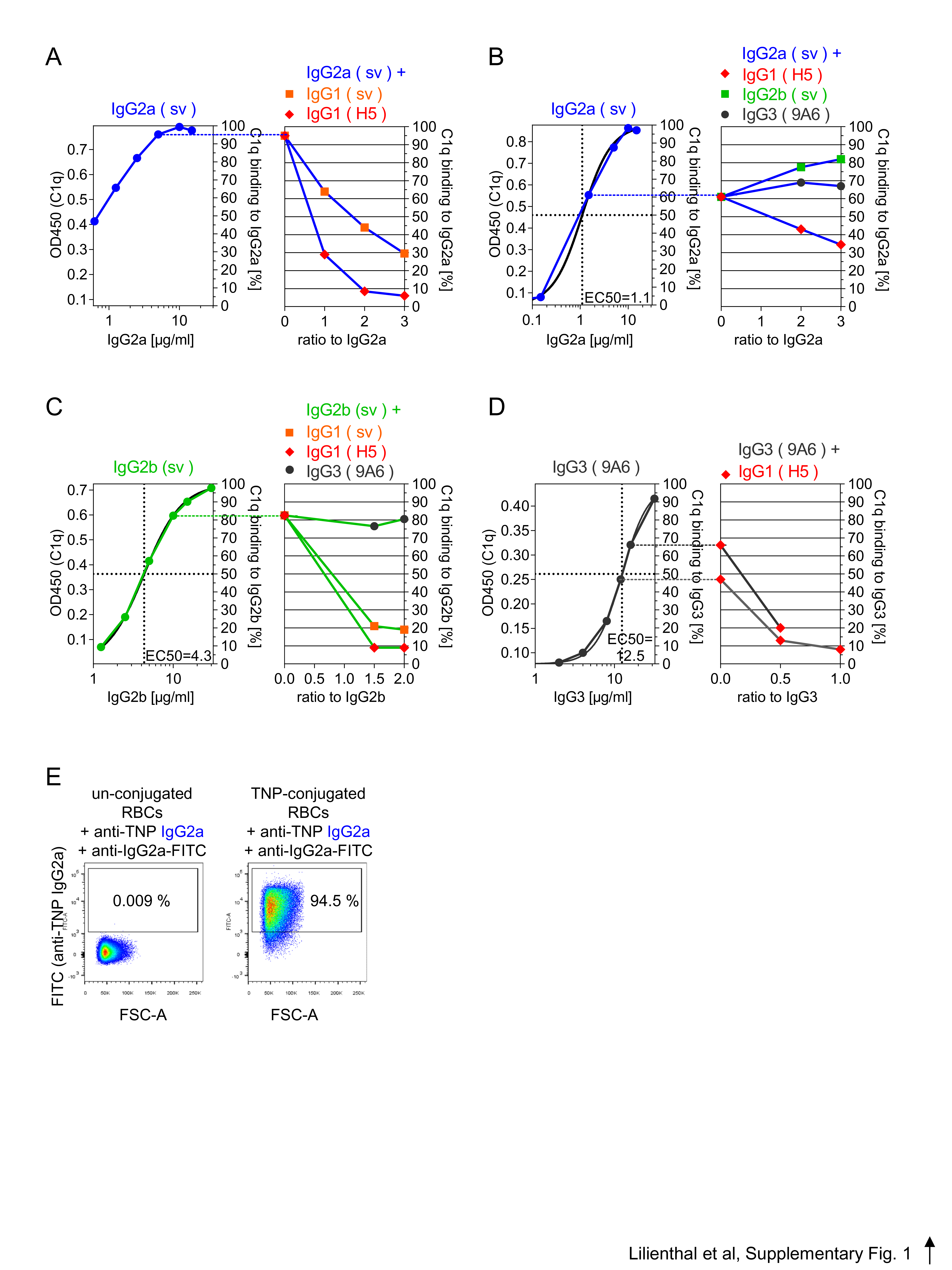

Supplement: Figure S1 — Inhibition of C1q binding to IgG2a, IgG2b, and IgG3 by IgG1. (A–D) C1q ELISA experiments as described and partially shown in Figures 1A–C with 2,4,6-trinitrophenyl (TNP)-Ficoll-coated 96-well plates, which were incubated with different concentrations of one or two anti-TNP monoclonal IgG subclass Abs [anti-TNP IgG1 (clone H5; red), IgG3 (clone 9A6; gray) as well as IgG1 (orange), IgG2a (blue), and IgG2b (green) class-switch variants (sv; with identical VDJ sequences)] and subsequently with serum containing C1q that was detected with an anti-C1q-HRP-coupled secondary Ab system. The left figure parts in panels (A–D) show the single IgG2a (blue), IgG2b (green), or IgG3 (gray) OD 450 nm (OD 450; left y-axes; each point: mean of n = 2) value binding curves and their interpolated binding curves with the percentage (right y-axes) of their maximal C1q binding [black; R2 of interpolation was >0.99; the top of the (interpolated) IgG subclass-specific curves was set to 100%, and the bottom of the curves was set to 0%]. The IgG subclass-specific half-maximal effective concentration (EC50) was calculated from the interpolated C1q-binding curves. To the indicated (dashed line) amounts of single IgG subclasses [(A) 5 µg/ml IgG2a; (B) 1.5 µg/ml IgG2a; (C) 10 µg/ml IgG2b; (D) 12 or 16 µg/ml IgG3], the indicated x-fold amounts of a second indicated anti-TNP IgG subclass were added. Their resulting OD 450 values and calculated percentages are presented in the right figure parts. (E) Verification of the TNP-coupling to red blood cell (RBC) as described in Figure 1D. TNP coupling to RBCs was verified by flow cytometry with murine anti-TNP IgG2a (sv) and FITC-labeled anti-murine IgG2a Abs. The results from one of at least two independent experiments are presented. [file Image_1.tif]
